# Health Volunteers Overseas: A Model for Ethical and Effective Short-Term Global Health Training in Low-Resource Countries

**DOI:** 10.9745/GHSP-D-19-00140

**Published:** 2019-09-23

**Authors:** Elizabeth MacNairn

**Affiliations:** a Health Volunteers Overseas, Washington, DC, USA.

## Abstract

Three core attributes enable short-term volunteers to make incremental contributions to long-term outcomes at host institutions: (1) focusing on teaching rather than service delivery, (2) engaging in mutually beneficial and equitable partnerships with host institutions, and (3) operating within a structured management system.

## INTRODUCTION

Health care professionals from high-income countries are increasingly interested and engaged in short-term volunteer global health experiences in low-resource settings.^1^ These experiences may focus on direct service delivery, teaching and training, humanitarian relief work, or global health electives for medical students and residents, among other purposes. A growing body of academic literature addresses guidelines for ethical global health engagement, but there is little consensus on standards and scant evidence of the benefits and potential harms of the engagement efforts on host institutions and communities.^2^ Additionally, much of the existing literature centers on the perspectives of “sending” institutions and clinicians, rather than the viewpoints and priorities of “host” institutions and practitioners.^3^

A wide variety of sending institutions engage in short-term global health work. These institutions include charities, churches, or other faith-based organizations, universities, and for-profit entities, and each employs defined operational models to achieve organizational objectives. For example, in the “fly-in medical mission” model, individuals or teams of health professionals volunteer to travel to underserved communities to provide dental care or health services that are otherwise unavailable, such as cleft palate repair or cataract surgery.^4^ While this model may deliver needed health services, it comes at a high financial cost, estimated at US$3.7 billion annually.^4^ Other potential problems associated with a direct service delivery approach, such as the fly-in medical mission model, include a high burden on host institution staff in resource-limited settings, power imbalances and perpetuation of global health inequities, a lack of bilateral participatory relationships and longitudinal planning, and concerns about long-term sustainability and patient safety.^5^

In this article, I argue that a well-designed and ethical global health engagement model that combines effective volunteer management systems with mutually beneficial partnerships can maximize the potential benefits and minimize the costs, or harms, of such programs. Expanding on an article published in 2017 describing the Health Volunteers Overseas (HVO) partnership model,^6^ I discuss HVO's health workforce capacity building approach in low- and middle-income countries (LMICs), focusing on the organization's unique short-term volunteer management structure, while considering its strengths, constraints, and implications.

HVO is a U.S.-based nonprofit organization founded in 1986 that aims to improve the quality and availability of health care in LMICs through teaching, training, and professional mentorship of the local health workforce. HVO deploys a short-term, highly skilled volunteer model to achieve its mission. Through HVO, more than 6,400 volunteer health professionals have completed an estimated 11,500 short-term assignments in 55 countries, serving in 248 different training projects. Table 1 shows a summary of HVO's 2018 activities.

**TABLE 1.** Health Volunteers Overseas: 2018 in Numbers

	Number
Volunteers	411^a^
Volunteer assignments	449
Volunteers who completed 2 or more assignments (January 1 to December 31, 2018)	38
Projects	101
Volunteer days served	7,169
Local health professionals who received training and mentorship	≥3,000
Clinical areas	18
Countries	23
Volunteer leaders	228
Collaborating institutions	90

a 40% had previously volunteered with HVO.

## RATIONALE

### Global Health Workforce Shortage

There is a global shortage of health care providers, and it is estimated that it will reach 18 million by 2030.^7^ This deficit disproportionately affects LMICs, where the burden of disease is highest and the ability to educate and support the health workforce is limited. These environments do not have enough health care professionals, few of the professionals have the opportunity for continued professional education or subspecialty training, and many work in isolation with large patient loads and limited resources. The “brain drain,” in which clinicians from resource-poor countries emigrate to higher-income countries, further exacerbates the shortage. Health worker performance in LMICs also remains a challenge to delivering high-quality, evidence-based health care.^8^ Yet, a well-trained and appropriately deployed global health workforce is essential to achieving universal health coverage, economic growth, and the United Nations Sustainable Development Goals.^9^^–^^11^

### Standards for Short-Term Global Health Engagement

Guidelines for short-term global health engagement typically start with the principles of beneficence and nonmaleficence,^12^ or “do no harm,” while also enumerating certain principles or recommendations. For example, a 2018 *American College of Physicians* paper identifies 5 positions, including predeparture preparation as an ethical requirement, in and of itself.^12^ A 2017 *Pediatrics* article summarizes 10 recommendations for trainee and clinician preparation, including exploring personal motivations, avoiding “poverty tourism,” and ensuring that professional goals are clarified and aligned with host goals. The authors call for the establishment of preparation standards in partnership with both sending and host institutions.^13^

Guidelines for short-term global health engagement contain principles of beneficence and nonmaleficence.

An article from the host institution perspective, published in *BMJ Global Health*, addresses expectations for international visiting faculty including developing mutually agreed-upon goals and careful selection and preparation of guest faculty to meet host's goals. The authors give the following instruction^14^:

*You plan the time the person should be here and when he/she is needed most, and you plan the curriculum and the topics. So they also come prepared and send someone who is an expert.*

Research from a 2018 *Globalization in Health* study defines the following 6 core consensus principles for effective and ethical short-term global health activities: (1) appropriate recruitment, preparation, and supervision of volunteers; (2) host partner, who defines the program and their role in it; (3) sustainability and continuity of programs; (4) respect for governance, legal, and ethical standards; (5) regular evaluation of programs for impact; and (6) mutuality of learning and respect for local health professionals.^1^

The next section of this article focuses on how the HVO short-term global health engagement model operationalizes best practices, similar to the guidelines discussed above, in a systematized, effective, and financially sustainable approach. This approach has been replicated and adapted within HVO itself, enabling the organization to grow from initially addressing just 1 area of clinical training, orthopedics, 33 years ago to 18 different clinical areas today (Table 2). This model can be implemented, in whole or in part, by other organizations or entities seeking to address global health training needs in cost-effective and ethical approaches.

**TABLE 2.** Health Volunteers Overseas' Clinical Training Areas and Current Project Sites (2018)

Clinical Area	Current Project Sites
Anesthesia (MD and nurse anesthesia)	Bhutan, Cambodia (Kampot, Siem Reap), Ghana (Kumasi, Tamale), Laos, Malawi, Rwanda, Vietnam (Ho Chi Minh City, Hue)
Dermatology	Cambodia, Costa Rica, Nepal, Uganda, Vietnam
Hand surgery	Ghana, Honduras, Nicaragua^a^
Hand therapy	Ghana, Nicaragua^a^
Hematology	Cambodia, Peru, Tanzania, Uganda
Internal medicine	Bhutan, Cambodia (Kampot, Phnom Penh), Costa Rica, Guyana, India, Nepal, Uganda (Kabale, Kampala, Mbarara)
Nursing education	Cambodia (Phnom Penh, Siem Reap), Laos, Tanzania, Uganda, Vietnam (Hai Duong, Hue)
Obstetrics and gynecology	Cambodia, Haiti, Uganda, Vietnam
Oncology	Bhutan, Honduras, Nepal (Bhaktapur, Kathmandu), Uganda, Vietnam
Oral health	Haiti, Laos, Nepal, Peru, Tanzania
Orthopedics	Bhutan (Mongar, Thimphu), China, Costa Rica, Ghana, Myanmar, Nicaragua,^a^ Philippines, St. Lucia, Tanzania, Uganda
Pediatrics	Bhutan, Cambodia, Laos, Nepal, Nicaragua^a^, St. Lucia, Uganda (Kampala, Kabale)
Physical therapy	Bhutan, India, Malawi, Rwanda, St. Lucia, Vietnam (Da Nang University of Medical Technology and Pharmacy, Da Nang Orthopedic and Rehabilitation Hospital)
Wound and lymphedema	Cambodia (Phnom Penh, Siem Reap), Haiti, India
Other project areas	Emergency medicine: Bhutan, Cambodia
	Mental health: Bhutan
	Residency training: Bhutan
	Pharmacy: Uganda

a Nicaragua activities suspended due to political unrest.

## THE HVO MODEL

### Overview

HVO programs address both the shortage of health workers and the quality of care delivered in LMICs by teaching, training, and mentoring health care providers, including faculty, residents, and students based in hospitals, clinics, and universities (Supplement 1). Since its inception, HVO has focused on teaching rather than service delivery, although its St. Lucia site centers on clinical care provision. To achieve its mission, HVO sends highly skilled, short-term volunteers to teach in projects that are designed with host partners and articulate clearly defined goals and objectives, as well as monitoring and evaluation processes. The organization's health worker capacity building approach is distinguished by 3 core attributes: efforts are education focused, volunteer driven, and partnership based.

**Figure:**
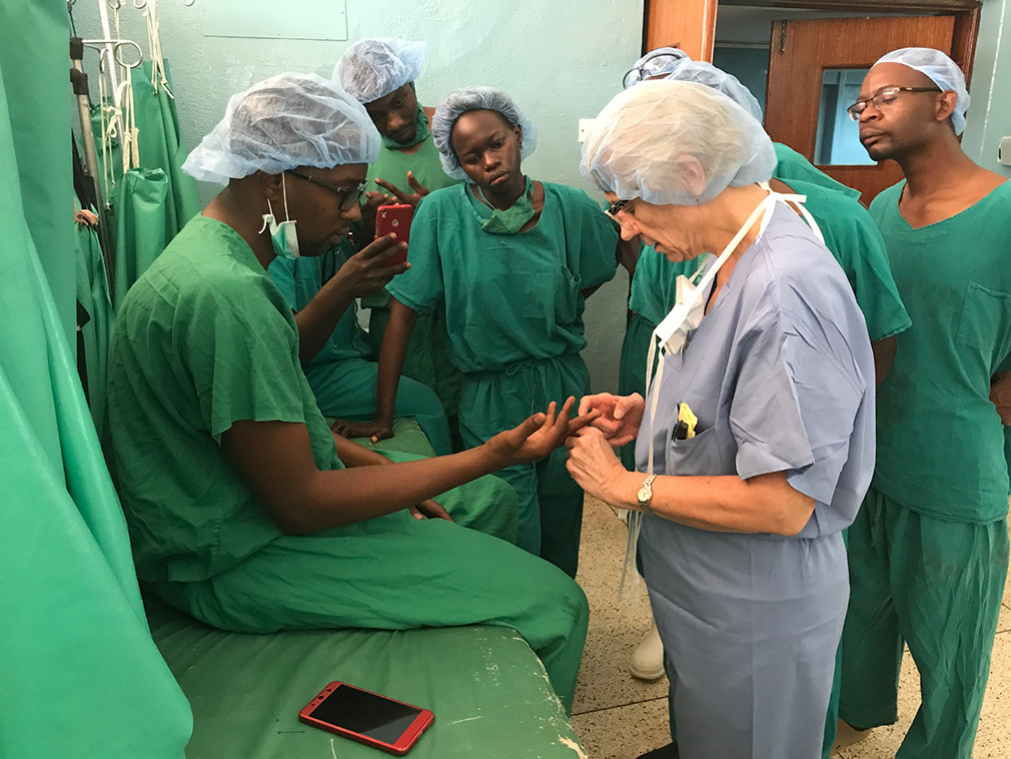
A volunteer provides training in hand surgery at Health Volunteers Overseas' project in Kampala, Uganda. **©** 2018/Health Volunteers Overseas

The project promotes lifelong learning, partners with host institutions, and identifies and trains local personnel.

- **Education focused:** HVO is committed to providing education that builds health worker capacity and promotes exchange of knowledge and skills between health care provider peers. It develops partnerships with host institutions that address their long-term training needs and priorities. Project design is guided by local diseases and conditions, and the projects are relevant and realistic, focus on prevention (when appropriate), promote lifelong learning, and identify and train local personnel who will assume the roles of both educator and provider.
- **Partnership based:** HVO establishes ethical and mutually beneficial partnerships with host institutions—hospitals, clinics, and universities—to provide education, training, and mentorship. A set of key principles serves as the foundation of HVO's partnership model, including mutual goal setting, honest and open communication, equity, mutual benefit, active partner engagement throughout the project life cycle, flexibility, and clearly defined leadership roles. Another key component is identification of a local champion who can inspire and motivate others, help maintain project momentum, and guide partnership evolution.^6^
- **Volunteer driven:** Project implementation is ensured by a small staff and a large cadre of volunteer health professionals, based both in the United States and abroad, who serve in clearly delineated roles. Annually, an estimated 600 volunteer health professionals serve with HVO as teachers, project directors, steering committee members, or on-site coordinators, supporting more than 90 projects in approximately 25 countries. Roles are defined in *HVO's Leadership Manual* and *Guide to Starting New Projects* as well as in agreement letters signed with each site when projects are established.

### Human Resource Infrastructure

The HVO model has a unique and cost-effective human resource infrastructure that assures project management and oversight, enabling effective short-term global health engagement. Key stakeholders include staff, volunteers, project directors, on-site coordinators and steering committee members.

The HVO model has a unique and cost-effective human resource infrastructure that assures project management and oversight.

Staff administer the volunteer management system, implementing processes to ensure timely and on-target project activities. Staff serve as the communications, coordination, and support nexus for the organization.

Volunteers are fully licensed health care professionals from private practice and academic settings who teach, train and mentor students, residents, faculty, and other health care providers in 2-week to 1-month assignments, depending on the site. The annual number of volunteers needed at each project site is assessed based on feedback from clinical leadership. HVO strives to identify and send the requested number of volunteers and measures progress toward recruitment goals annually.

Some HVO project sites can also accommodate residents, if they are accompanied by a preceptor. Volunteers are responsible for funding their own airfare and accommodations although HVO has several grant opportunities to defray these costs including the Orthopaedics Traveling Fellowship and the Plotnick Nursing Education Volunteer Fund. Approximately 28% of 2018 volunteers received partial funding to support their overseas assignments.

Project directors are experienced health professionals whose responsibilities include project design and monitoring, volunteer selection and orientation, and technical oversight. They help prospective volunteers set realistic expectations, understand site needs, and thoughtfully prepare for assignments. Project directors submit annual surveys outlining accomplishments, challenges, and new initiatives, and they are expected to be in regular contact with their sites, including visiting, to stay abreast of important changes and issues.

On-site coordinators are selected by host partner institutions for each project. Some HVO project sites identify both an administrative as well as a clinical coordinator, who is typically a senior faculty member or department head. Administrative coordinators assist with visas, arrival arrangements, and housing issues, liaising closely with HVO staff. Clinical on-site coordinators identify training needs; approve, schedule, and orient volunteers; and provide feedback on both volunteers and evolving project needs through informal and formal mechanisms, including an annual survey. Their level of engagement depends on individual motivation and availability and thus varies from project to project, which can affect project quality.

Steering committees provide programmatic oversight for each of the 18 clinical divisions. Each steering committee is composed of 3–10 volunteer health professionals who approve new projects and review existing projects (and, when appropriate, suspend or close projects); set policy on the types of volunteers who can be placed; help to recruit new members and volunteers; and plan workshops, meetings, and other outreach activities. Steering committee members are selected with input from HVO staff and current committee members, based on criteria such as commitment to teaching and training, availability to participate in 1 or 2 meetings per year, and global health experience.

HVO volunteers, project directors, on-site coordinators, and steering committee members are volunteers who donate their time and expertise to the organization, receiving no compensation for their efforts. In 2018, the value of services donated to HVO totaled approximately $6,743,500.

In 2018, the value of services donated to HVO totaled nearly US$7 million.

### Volunteer Management System

Over 3 decades, HVO has developed —and continues to refine—a volunteer management system that enables highly skilled health professionals to make incremental but sustained improvements in the availability and quality of care delivered in low-resource settings. Each volunteer's teaching assignment contributes to stated project goals and objectives and builds upon the previous assignment. For example, a team of 5 HVO volunteer nurse educators worked with nurses at Hue University of Medicine and Pharmacy in Vietnam who wanted to expand their research capacity. Together, the nurses developed a 2-year plan for their research agenda, which included education about research methodologies, grant writing, and publishing in peer-reviewed journals. Each HVO volunteer nurse educator knew what she was expected to teach to advance the collaborative plan, and the Vietnamese nurses were confident in achieving their goals and advocating for needed resources.

In any given year, 40% of volunteers have completed a previous HVO assignment. Some return to the same site, providing teaching continuity, while others volunteer at new sites. Repeat volunteers can also share insights and techniques learned at one site and bring this information to new sites. In this way, HVO's short-term volunteers help expand a network of professional peers who sometimes continue to engage long after a volunteer leaves the host institution.

HVO is sponsored by 18 professional associations (Supplement 2), including the American Academy of Pediatrics, the American Society of Clinical Oncology, and the American Physical Therapy Association. These associations provide invaluable access to their membership, which serves as a primary volunteer pipeline for our projects, while HVO provides sponsors with structured teaching and training assignments and the opportunity for their members to participate directly in their specialty's global health community.

### Key Processes

HVO's volunteer management system includes 4 key processes, as described below. An array of resources support these processes, ensure quality implementation, and enable organizational learning. These HVO resources include the comprehensive *Guide to Volunteering Overseas*, *Leadership Manual*, *Guide to Starting New Projects*, and *KnowNET*, a password-protected intranet for volunteers and project directors that provides volunteer schedules, teaching resources, and policies on research and donations of equipment and pharmaceuticals.

#### Volunteer Application, Vetting, and Approval

Potential volunteers complete an online application and provide a curriculum vitae (CV), which HVO staff review to determine if applicants have appropriate credentials to participate in projects. Staff contact applicants to learn more about their motivations for volunteering, commitment to training and teaching, availability for assignment, and geographical or programmatic preferences. Applicant CVs are then shared with relevant project directors and on-site coordinators for review and additional follow-up, ensuring that all applicants are vetted by peer health care professionals with the technical skills and global health experience to evaluate their capacity to engage productively in HVO training projects. Before a final decision is made, project directors check applicant references.

#### Volunteer Scheduling, Planning, and Logistics Support

HVO staff work with approved volunteers to schedule assignments based on site needs and timelines, and volunteer availability. Most volunteers serve as individuals, although HVO coordinates a limited number of team assignments for multidisciplinary projects (e.g., spine surgery, wound management, oncology) when appropriate. HVO teams are usually composed of 3 or 4 volunteers.

HVO staff provide significant planning and logistics support to volunteers throughout their engagement, significantly reducing the burden on both host institutions and the volunteers themselves. Increasingly, for example, host country ministries of health require visiting clinicians to register with national medical and nursing associations. Such processes are important but also time-consuming, requiring background checks and the submission of multiple documents.

HVO staff provide detailed instructions, help volunteers fulfill credentialing and registration requirements, and liaise with host personnel and volunteers throughout the process. Staff also work with volunteers to address assignment logistics and orientation, which includes obtaining visas; assisting with questions about flight, hotel, and arrival arrangements; providing cultural, political, and historical information; and providing information on personal health and safety.

#### Professional Orientation (Predeparture and On-Site)

Predeparture and on-site orientation for volunteers is provided by a network of HVO stakeholders, including staff, project directors, on-site coordinators, and, often, previous volunteers. Information, shared through email, phone, and Skype exchanges, addresses site training needs and priorities, partner institution structure, prospective trainees and their educational levels, prevalent local diseases and conditions, locally available resources (e.g., pharmaceuticals, diagnostic testing), and previous volunteers' presentations and evaluations.

Predeparture orientation may also include identification of specifically requested lecture topics to ensure that assignments align with host priority training needs. The *HVO Guide to Volunteering Overseas* and *KnowNET* (the HVO intranet for volunteers and clinical leadership) provide a range of information to help volunteers prepare for their assignments, both professionally and personally. Once a volunteer arrives on-site, host faculty typically provide a short briefing, although the responsibility for professional integration rests primarily with the volunteer. HVO emphasizes that it is essential for volunteers to prepare as fully as possible for their assignments prior to departure because site-based staff are busy with their regular (and usually very high) patient loads, teaching, and family responsibilities as well as second jobs in some cases.

Dr. Jon Kolkin, an HVO orthopedics volunteer, summed up the personal qualities essential for successful global health volunteers^15^:

*… humility, compassion, patience and flexibility. … One must be willing to think creatively, look at a situation from multiple viewpoints and adopt therapeutic strategies to accommodate and respect local conditions, cultures, techniques, politics, resources, educational backgrounds, demographics and social norms.*

#### Volunteers' Evaluations and Recognition

Returned volunteers and multidisciplinary teams complete an online survey to evaluate several dimensions of their teaching and overall experience. Repeat volunteers to the same site complete a slightly different survey, which elicits their assessment of HVO's longer-term impact on the site. As needed and on a continuous basis, HVO staff reach out to volunteers to discuss their feedback surveys and share surveys with clinical leadership to address immediate problems and make needed improvements in project implementation.

On an annual basis, project directors and on-site coordinators complete a survey assessing project achievements, challenges, and needed changes in project design. HVO staff collate and review these data, sharing with steering committees, project directors, and on-site coordinators to inform adaptations in project design and identify and address challenges and opportunities (Table 3 and Table 4).

**TABLE 3.** Observations of On-Site Coordinators on General Areas of Improvement as a Result of Health Volunteers Overseas' Activities, 2018 Survey (N=65)^a^

Observations	% of On-Site Coordinators
Improvements in staff skills	91
Improvements in staff attitudes	88
Increased efficiency of care	86
New techniques introduced and utilized	85
Improvements in patient outcomes	84
Improvements in patient safety	82

a 73% response rate.

**TABLE 4.** Observations of On-Site Coordinators on Improvements Due to Health Volunteers Overseas Training, 2018 Survey

Country	Clinical Specialty	Observed Improvement/Accomplishment
Bhutan	Nursing/oncology	Development and implementation of nursing chemotherapy assessment form that was approved and implemented by nursing department as a standard of care practice
Bhutan	Emergency medicine	Initiation of emergency medicine residency program and emergency medical technician/emergency medical responder program
Cambodia	Anesthesia	Epidural analgesia protocol implemented in maternity department
Ghana	Hand therapy	Implementation of new protocol for management of flexor tendon repairs
Haiti	Physical therapy	Clinical guidelines and assessment form for stroke patients implemented
Nepal	Oral health	Clinical protocols in orthodontics implemented, and adoption of conscious sedation for the first time in the dental department
Peru	Hematology	Introduction of the Wright-Giemsa stain for better cytological evaluation of bone marrow smears in hematological diseases, new to hospital
Tanzania	Hematology	Clinical protocols developed for management of all hematological malignancies

HVO recognizes outstanding volunteers—based at both host institutions and those who travel to teach—with the annual Golden Apple Award for exceptional contributions to HVO's mission. Recognizing exceptional service and commitment is essential in a volunteer-driven organization like HVO because it expresses appreciation, establishes organizational role models, and encourages ongoing engagement.

## DISCUSSION

### Strengths of HVO's Model

HVO's model has important strengths that enable short-term volunteers to make incremental contributions to long-term outcomes at host institutions. The human resource infrastructure of this model is a unique combination of administrative staff and volunteer technical leadership based both in the United States and at host institutions who work together to support volunteer teachers and ensure quality programming and sustained impact. Strengths of the HVO model include effective systems to vet, approve, and prepare volunteers; opportunities for health leadership development and recognition; the opportunity to develop enduring professional relationships; sustainability, cost efficiency, and replicability across a breadth of clinical specialties; and mutual skills transfer.

**Figure:**
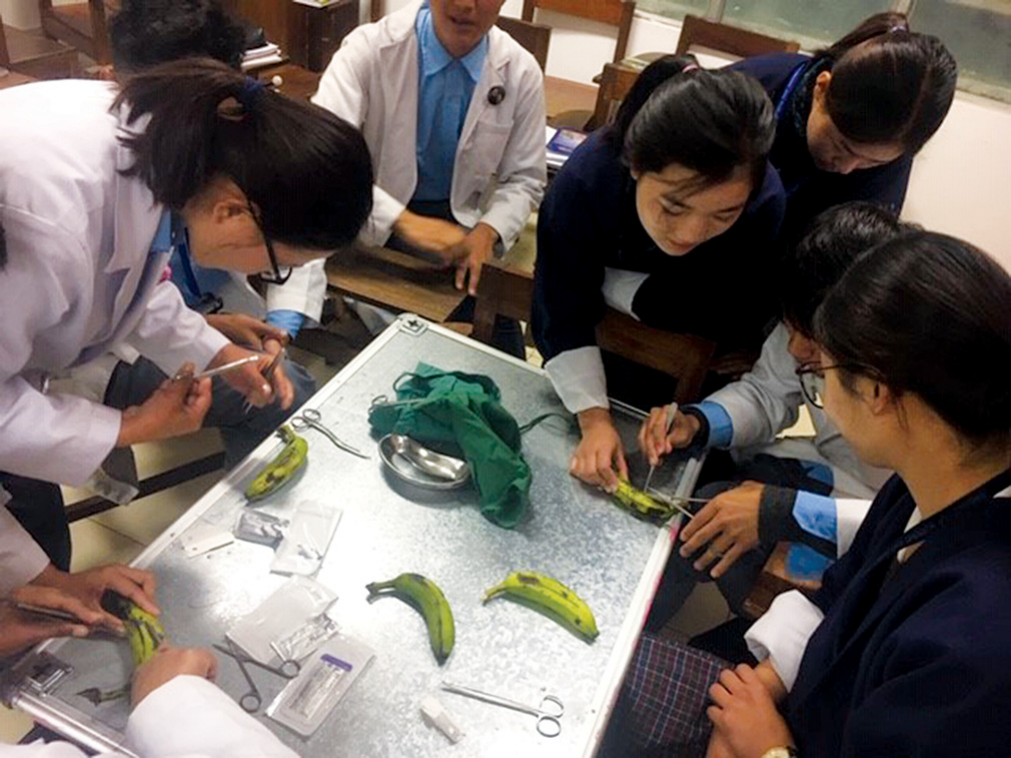
Clinicians learn to suture at Health Volunteers Overseas' emergency medicine project in Thimphu, Bhutan. © 2018/Health Volunteers Overseas

HVO's model has important strengths that enable short-term volunteers to make incremental contributions to long-term outcomes at host institutions.

#### Volunteer Management Systems

As previously described, HVO has detailed volunteer management processes to ensure that qualified and committed volunteers with appropriate general or subspecialty expertise are selected for projects, well-prepared for their assignments, and scheduled according to site needs as much as is possible. These systems enable effective and ethical short-term global health engagement.

#### Cost Efficiency, Sustainability, and Replicability Across Multiple Clinical Specialties

While each project differs according to host needs and priorities, HVO has well-documented and efficient systems for partnership development and program monitoring that promote both sustainability and replicability, enabling HVO to explore new countries, clinical specialties, and educational approaches. HVO's annual operating budget is approximately $1 million, primarily from individual donors, which enables a core staff of 12 to support clinical leadership, volunteers, and project sites. HVO's clinical expertise and leadership are provided by a cadre of more than 600 volunteer health professionals annually, which reduces costs significantly while also providing sustained support for the training of local health care providers.

#### Opportunities for Leadership Development

Throughout the organization's structure, volunteers fulfill a variety of roles to design, implement, and monitor training projects and to support other volunteers. HVO continuously seeks new leadership to fulfill these roles, offering opportunities for clinicians to broaden their professional experience.

#### Long-Term Collaborations

Strengthening health systems through education and training is a long-term endeavor. Health professionals in both LMICs and higher-income countries alike need ongoing opportunities to hone their skills and learn new approaches, integrate new knowledge into their practice, and develop professionally. HVO establishes enduring partnerships with host institutions, some of which have lasted 3 decades or more, enabling the growth of mutually beneficial and trusting relationships, the creation of a peer professional network, and promotion of opportunities for ongoing training, conference attendance, and research collaboration.

#### Mutual Skills Transfer

Many HVO volunteers report they learn more than they teach, citing in particular the opportunity to learn about unfamiliar or advanced stage diseases and health conditions, and the provision of care in resource-constrained environments lacking diagnostic tests, advanced equipment, medicines, and sufficient staff.

### Constraints of the HVO Model

The types of potential ethical dilemmas inherent in the deployment of short-term health volunteers to LMICs are well-documented and are exactly the dilemmas that our volunteer management model are designed to mitigate, to the extent possible. The HVO model does present some specific constraints that merit exploration, including the following issues.

#### Data Limitations

HVO's monitoring and evaluation system, including the data collection tools discussed above, effectively captures project-level data on achievements, challenges, and evolving needs. However, it is difficult to isolate the impact of either an individual HVO volunteer or HVO as a whole on a site, given the range of training inputs, including country-led expertise and the capacity building support provided by other outside actors, such as NGOs, universities, and hospitals. During the next 2 years, HVO plans to develop several in-depth case studies to capture the impact of training efforts over time and to implement a trainee-specific survey.

While HVO's model is designed to foster progressive and sustained improvements, rapid and measurable gains in evidence-based patient care sometimes occur. In 2018, for example, a volunteer pediatric intensivist and pediatric resident in a Bhutanese hospital worked together to explore high mortality levels in infants in the pediatric intensive care unit (PICU) that had been attributed to acute encephalopathy. The clinicians postulated and later demonstrated that the infants had thiamine deficiency linked to infantile beriberi, leading to new standards of care in the PICU of that hospital and others in Bhutan. Initial data from their study showed that thiamine administration to these children led to a precipitous drop in infant mortality in the PICU (Box).

BOX **Case Study: Identifying Infantile Beriberi in Bhutan**

*This case study is a condensed version of a longer article published in the HVO summer 2019 newsletter, The Volunteer Connection, written by Dr. Dinesh Pradhan, Pediatric Resident, Khesar Gyalpo University of Medical Sciences of Bhutan (Thimphu, Bhutan), and HVO volunteer Dr. Christoph Funk, Pediatric Intensivist, Dietrich-Bonhoeffer-Klinikum (Neubrandenburg, German), with contributions from Dr. Leila Srour, Chair of HVO's Pediatric Steering Committee. Dr. Funk served in a 3-month volunteer assignment in Bhutan in 2018, working closely with Dr. Pradhan and other pediatric care providers at the project site.*

Bhutanese pediatricians at the National Referral Hospital in Thimphu were grappling with a perplexing problem in the pediatric intensive care unit (PICU): infants who initially presented with nonspecific respiratory or gastrointestinal symptoms that rapidly progressed to acute encephalopathy and, within a week, led to death in almost 80% of cases. Survivors had serious neurological sequelae.

Management focused on treating them as “meningoencephalitis” cases with a possible viral etiology. They were treated with antibiotics, antivirals, anti-epileptics, and general supportive care, including nutrition, hydration, and ventilator support, with poor results. Collaboration with the National Institute of Virology in India and the Centers for Disease Control and Prevention in the United States to isolate a virus from the cerebrospinal fluid of these children was not successful.

After observing the survival of such a patient following Dr. Pradhan's administration of a cocktail of multivitamins, Dr. Funk analyzed the case, postulating that thiamine could have been the key ingredient that made the difference. He researched the literature, which pointed to the possibility of these cases being “infantile beriberi” or thiamine deficiency.

Supported by the hospital's pediatrics department, Drs. Pradhan and Funk sought to prove their hypothesis. They adapted a protocol from an Indian study to administer thiamine to these children and observe for any improvements, collecting 1 year of data (January–December 2018). They compared 19 children who had not received thiamine (January–July) with 32 who had received it (August–December). None of the children in the thiamine cohort died, whereas 73.7% in the no-thiamine cohort had died. The doctors will seek to prove their empirical findings, but based on their initial study, thiamine administration to children with acute encephalopathy is now standard-of-care in the PICU.

Dr. Pradhan (with funding from HVO) and Dr. Funk presented their research on infantile beriberi in Bhutan at the 2019 Annual Conference of the German Society of Tropical Paediatrics and International Child Health (GTP) and won the Helmut Wolf Award for their work, selected by a jury of scientists and clinicians.

#### Teaching Continuity

In HVO's global health capacity building model, ensuring continuity between volunteer assignments is an ongoing challenge. Aligning the availability of a subspecialty volunteer with a host institution semester-long curricula, for example, can be difficult. This kind of scheduling challenge can be mitigated through advanced and thoughtful planning and, potentially, a combination of on-site and remote teaching. Similarly, ensuring continuity of educational content between volunteers introduces a complexity that is not always surmountable, although in strong collaborations between on-site coordinators and project directors, this risk can be mitigated.

Repeat volunteers are invaluable to host sites because they can provide needed continuity, orient first-time volunteers, build and enhance trusting professional relationships with host institution personnel, and develop a deep understanding of site priorities. Sharing lectures and presentations through HVO's KnowNET is another way of promoting continuity of training.

#### The “Failed Volunteer”

Although it is rare, some HVO volunteers are unsuccessful. Typically, these individuals are volunteers who, despite preparation, realize that they are not personally or professionally equipped to work effectively or to manage the stress of resource-limited environments. They may unduly burden hosts or may interact in a disrespectful or unproductive manner with host institution students or faculty. Such volunteers are usually quickly identified by on-site coordinators or by other HVO volunteers serving at the site. Depending on the circumstances, HVO may not allow the individuals to volunteer with the organization again.

HVO's intensive volunteer vetting, approval, and preparation processes tend to screen out such individuals, but it is not always able to do so. We have occasionally found that unsuccessful first-time volunteers can become successful volunteers through the transformative experience of their work with HVO. The capacity for and practice of personal reflection are key in these circumstances because the individual can ultimately achieve “transformation, meaning and connection”^16^ through experience.

## FUTURE IMPLICATIONS AND CONCLUSION

HVO presents a replicable model for ethical and effective short-term global health experiences. Over more than 30 years, HVO has developed efficient and comprehensive volunteer management structures and systems that enable highly skilled volunteers to improve health workforce capacity in LMICs through short-term teaching and training assignments. HVO's model integrates best practice guidelines for short-term global health engagement with well-designed training projects implemented through long-term, equitable, and mutually beneficial partnerships.

HVO presents a replicable model for ethical and effective short-term global health experiences.

An effective and ethical volunteer management system also enables HVO to explore new approaches for education and training delivery that build upon the existing platform. In 2015, HVO launched the Wyss Scholarship for Future Leaders in Global Health to support the professional development and leadership skills of local health care providers at HVO project sites. Thus far, 30 scholars from a wide variety of clinical specialties and countries have been funded to attend conferences and participate in intensive training courses or observerships. In 2018, HVO sponsored the publication of an e-book entitled *International Partnerships for Strengthening Health Care Workforce Capacity: Models of Collaborative Education* in partnership with the open-access journal *Frontiers in Public Health*.^17^ The e-book consists of 33 peer-reviewed articles submitted by 163 authors from 28 different countries, representing 96 unique organizations and institutions.

Recent programmatic innovations include select opportunities for longer-term volunteers to design responses to complex global health training needs in collaboration with partner institutions, and e-volunteering or distance mentoring for activities such as curriculum development, research support, or leadership skills enhancement. New clinical areas have been added in recent years. For example, projects in obstetrics and gynecology started in 2017, and HVO is exploring a broader approach to physical rehabilitation to build upon the physical therapy program. Increasingly, HVO project sites request training in subspecialty areas such as neonatology, infectious disease, nephrology, cardiology, and neurology, as well as support for research. HVO is expanding recruitment to address these evolving site priorities.

HVO provides a well-structured and cost-efficient model for health professionals interested in sharing and building their skills and knowledge to improve and expand health care delivery in lower resource settings. HVO continues to refine and adapt our model to address evolving global health training needs at project sites and to ensure ongoing alignment with core principles of ethnical and effective global health engagement.

## Supplementary Material

19-00140-MacNairn-Supplement2.pdf

19-00140-MacNairn-Supplement1.pdf

## Notes

**Peer Reviewed**

**Funding:** No funding was received for this work.
