# Supplementary material for: Health Volunteers Overseas: A Model for Ethical and Effective Short-Term Global Health Training in Low-Resource Countries
Source: Glob Health Sci Pract. 2019 Sep 23;7(3):344–54. doi: 10.9745/GHSP-D-19-00140 (PMC6816812; doi:10.9745/GHSP-D-19-00140)
Supplement: 19-00140-MacNairn-Supplement2.pdf [file 19-00140-MacNairn-Supplement2.pdf]

MacNairn E. Health Volunteers Overseas: a model for ethical and effective short-term global health training in low-resource countries. *Glob Health Sci Pract*. 2019;7(3). https://doi.org/10.9745/GHSP-D-19-00140

SUPPLEMENT 2. Health Volunteers Overseas Professional Association Sponsors

- ADA Foundation
- American Academy of Dermatology
- American Academy of Pediatrics
- American Association for Hand Surgery
- American Association of Colleges of Nursing
- American Association of Nurse Anesthetists
- American Association of Oral and Maxillofacial Surgeons
- American College of Obstetricians & Gynecologists
- American College of Physicians
- American Foundation for Surgery of the Hand
- American Orthopaedic Foot and Ankle Society
- American Physical Therapy Association
- American Society of Clinical Oncology
- American Society of Hematology
- Association for the Advancement of Wound Care
- British Society of Haematology
- Oncology Nursing Society
- Society of Gynecologic Oncology
