# Supplementary material for: Health Volunteers Overseas: A Model for Ethical and Effective Short-Term Global Health Training in Low-Resource Countries
Source: Glob Health Sci Pract. 2019 Sep 23;7(3):344–54. doi: 10.9745/GHSP-D-19-00140 (PMC6816812; doi:10.9745/GHSP-D-19-00140)
Supplement: 19-00140-MacNairn-Supplement1.pdf [file 19-00140-MacNairn-Supplement1.pdf]

MacNairn E. Health Volunteers Overseas: a model for ethical and effective short-term global health training in low-resource countries. *Glob Health Sci Pract*. 2019;7(3). https://doi.org/10.9745/GHSP-D-19-00140

SUPPLEMENT 1. Health Volunteers Overseas Collaborating Institutions

BHUTAN
Mongar
Eastern Regional Referral Hospital
Thimphu
Jigme Dorji Wangchuck National Referral Hospital
Ministry of Health
Royal Institute of Health Sciences
University of Medical Sciences

CAMBODIA
Kampot
Sonja Kill Memorial Hospital
Phnom Penh
Ciomal Kien Kleng Centre
HOPE Medical Centers
Preah Kossamak Hospital
Sihanouk Hospital Center of HOPE
Siem Reap
Angkor Hospital for Children

CHINA
Wenzhou
Second Affiliated Hospital of Wenzhou Medical School

COSTA RICA
San Jose
Caja Costarricense de Seguro Social System
Costa Rican College of Physicians
University of Costa Rica

GHANA
Kumasi
Komfo Anokye Teaching Hospital
Tamale
University for Development Studies

GUYANA
Georgetown
Georgetown Public Hospital Corporation

HAITI
Deschapelles
Hôpital Albert Schweitzer
Port au Prince
Faculté d'Odontologie

HONDURAS
Tegucigalpa
Cancer Center Emma Callejas
Hospital Escuela
Hospital San Felipe

INDIA
Bangalore
Bangalore Baptist Hospital
Raxaul
Duncan Hospital
Vellore
Christian Medical College

LAOS
Luang Prabang
Lao Friends Hospital for Children
Vientiane
National University of Laos

MALAWI
Blantyre
Malawi College of Medicine
Queen Elizabeth Central Hospital
University of Malawi

MYANMAR
Yangon
Yangon General Hospital
Yangon Orthopaedic Hospital

NEPAL
Bhaktapur
Bhaktapur Cancer Hospital
Dhulikhel
Dhulikhel Hospital
Kathmandu
Dermatology Institute Skin Health and Referral Center
Kathmandu Medical College and Teaching Hospital
Kathmandu University
School of Medical Sciences
National Academy of Medical Sciences
Nepal Medical College and Teaching Hospital
Tansen
Lumbini Medical College

NICARAGUA
Managua
Hospital Escuela Antonio Lenin Fonseca
Hospital Escuela Roberto Calderón Gutierrez
Hospital Fernando Velez Paiz
Hospital Infantil Manuel Jesus Rivera
Hospital Militar Escuela Alejandro Davila Bolanos
Instituto Politecnico de la Salud

PERU
Arequipa
Catholic University of Santa Maria
Carlos Alberto Sequin Escobdo Hospital
Yanahuara Hospital
Chiclayo
The Craniofacial Center of Hospital Regional Docente, Las Mercedes

PHILIPPINES
Manila
Philippine General Hospital

RWANDA
Kigali
King Faisal Hospital
Rwanda Physiotherapy Association
University of Rwanda—School of Health Sciences, College of Medicine and Health Sciences

ST. LUCIA
Vieux Fort
St. Jude Hospital

TANZANIA
Dar es Salaam
Muhimbili National Hospital
Muhimbili University of Health and Allied Sciences
Tanzanian Dental Association
Moshi
Kilimanjaro Christian Medical Center
Mwanza
Weill Bugando Medical Centre

UGANDA
Kabale
Kabale Regional Referral Hospital
Kabale University School of Medicine
Kampala
Makerere University
Mulago National Referral Hospital
Mbarara
Mbarara University of Science and Technology
Mbarara University Teaching Hospital

VIETNAM
Da Nang
Da Nang Orthopedic and Rehabilitation Hospital
Da Nang University of Medical Technology and Pharmacy
Hai Duong
Hai Duong Medical and Technical University
Ho Chi Minh City
Hospital for Traumatology and Orthopaedics
Hue
Hue University of Medicine and Pharmacy
